# Age-associated mitochondrial DNA mutations lead to small but significant changes in cell proliferation and apoptosis in human colonic crypts

**DOI:** 10.1111/j.1474-9726.2009.00531.x

**Published:** 2010-02

**Authors:** Marco Nooteboom, Riem Johnson, Robert W Taylor, Nicholas A Wright, Robert N Lightowlers, Thomas B L Kirkwood, John C Mathers, Doug M Turnbull, Laura C Greaves

**Affiliations:** 1 Mitochondrial Research Group, Institute for Ageing and Health, Newcastle UniversityNewcastle upon Tyne NE2 4HH, UK; 2 Histopathology Lab, London Research Institute, CRUK London and Barts and the LondonUK; 3 Henry Wellcome Laboratory for Biogerontology Research, Institute for Ageing and Health, Newcastle University, Campus for Ageing and VitalityNewcastle upon Tyne, UK; 4 Human Nutrition Research Centre, Institute for Ageing and Health, Newcastle UniversityNewcastle upon Tyne NE2 4HH, UK

**Keywords:** aging, colon, mitochondrial DNA, respiratory chain, stem cells

## Abstract

Mitochondrial DNA (mtDNA) mutations are a cause of human disease and are proposed to have a role in human aging. Clonally expanded mtDNA point mutations have been detected in replicating tissues and have been shown to cause respiratory chain (RC) defects. The effect of these mutations on other cellular functions has not been established. Here, we investigate the consequences of RC deficiency on human colonic epithelial stem cells and their progeny in elderly individuals. We show for the first time in aging human tissue that RC deficiency attenuates cell proliferation and increases apoptosis in the progeny of RC deficient stem cells, leading to decreased crypt cell population.

Human aging is a complex process characterized by gradual decline in function and decreased ability to maintain tissue homeostasis. Acquired mutations of the mitochondrial genome have been proposed to contribute to human aging (Trifunovic, 2006). Mitochondrial DNA (mtDNA) encodes 13 essential polypeptides of the RC and is present in multiple copies within individual cells (Larsson & Clayton, 1995). When a somatic mutation occurs in an individual mtDNA molecule, functional complementation protects cells against biochemical defects (Nakada *et al.*, 2001). It is only when a mutation clonally expands to a critical threshold level that a biochemical defect is observed (Taylor & Turnbull, 2005).

We have shown previously that stem cells present in the base of colonic crypts accumulate high levels of clonally expanded mtDNA point mutations with age, leading to RC deficiency (Taylor *et al.*, 2003). The progeny of these stem cells populate the crypt (Wright, 2000). This provides an opportunity to determine if acquired mtDNA mutations in the stem cells alter the function of their progeny and contribute to the aging process.

In this study, we have investigated the consequences of RC deficiency in colonic crypt stem cell progeny using a dual immunofluorescent approach. The tissue used was histologically normal colonic mucosa taken from 15 human subjects undergoing resection for colorectal carcinoma. Each sample was at least 12 cm from the tumour site. Sections were cut, deparaffinized in Histoclear™ and rehydrated through a graded ethanol series. Antigens were retrieved by pressure cooking in 0.01 m sodium citrate (VWR) buffered to pH 6.0. Sections were washed and blocked in 1% normal goat serum (Sigma, Poole, UK) and endogenous biotin and avidin were blocked using a commercial kit where necessary (Vector Laboratories, Peterborough, UK). Sections were incubated in primary antibodies (Table 1) for 1 h at room temperature, washed in PBS + 0.1% Tween 20 and incubated for a further 30 min in an IgG subtype specific secondary antibody mix (Table 1), prior to further washing and mounting in Vectashield with DAPI (Vector Laboratories).

**Table 1:** Details of all antibodies used in double immunofluorescence experiments

Primary antibody	Ki-67 (DAKO, Cambridge, UK)	M30CytoDeath (Roche)	β-Catenin (BD Biosciences, Oxford, UK)	Cytochrome *c* oxidase subunit I (Invitrogen, Paisley, UK)
Function under investigation	Cell proliferation	Apoptosis	APC pathway	Respiratory chain
Antibody subtype	IgG_1_	IgG_2b_	IgG_1_	IgG_2a_
Concentration	0.5 μg mL^−1^	Manufacturer recommended	1.25 μg mL^−1^	5 μg mL^−1^
Secondary antibody	Goat anti-mouse IgG_1_ rhodamine conjugated (Jackson ImmunoResearch, Westgrove, PA, USA)	Goat anti-mouse IgG_2b_ biotinylated (Jackson ImmunoResearch)	Goat anti-mouse IgG_1_ rhodamine conjugated. (Jackson ImmunoResearch)	Goat anti-mouse IgG_2a_, Alexaflour conjugated (Invitrogen)
Tertiary antibody	N/A	Streptavadin–Rhodamine (Jackson ImmunoResearch)	N/A	N/A

We examined the replication of the progeny of stem cells using Ki-67 as a marker of cell proliferation. RC deficiency was defined as absence of expression of cytochrome *c* oxidase subunit I, which we have shown correlates with absence of enzyme activity (Taylor *et al.*, 2003) (Fig. 1A). Eighty-eight RC normal crypts and 56 RC deficient crypts were analysed from six subjects aged 73–89. Proliferation index in RC deficient crypts was evaluated by two-way anova, to adjust for subject to subject variation, and found to be significantly lower (*P* < 0.0001) than in normal crypts in all subjects [average decrease in proportion of proliferating cells – 16% (Fig. 1B)]. Crypt length was also recorded in this experiment (Fig. 1C). We investigated apoptosis in 201556 RC normal cells and 37166 RC deficient cells from 10 subjects aged 59–85 using an antibody to cleaved cytokeratin 18 [M30 CytoDeath (Roche, Burgess Hill, UK)] (Fig. 1D). Apoptotic frequency was significantly higher in RC deficient cells (0.36%) compared with normal cells (0.29%) using a two-way anova adjusted for subject variation [*F*(1,9) = 5.19; *P* = 0.049]. To investigate the structural consequences of the decrease in proliferation and increase in apoptosis we made an approximate measure of crypt cell population (Goodlad *et al.*, 1991). We calculated the mean number of cells in transverse sections of 25 RC normal and 25 RC deficient crypts for each of the six subjects investigated in the proliferation analysis, and multiplied this by the crypt lengths recorded above. RC deficient crypts had significantly fewer cells than normal crypts (anova, *P* < 0.0001, mean difference 17 ± 8%, Fig. 1E).

**Fig. 1:**
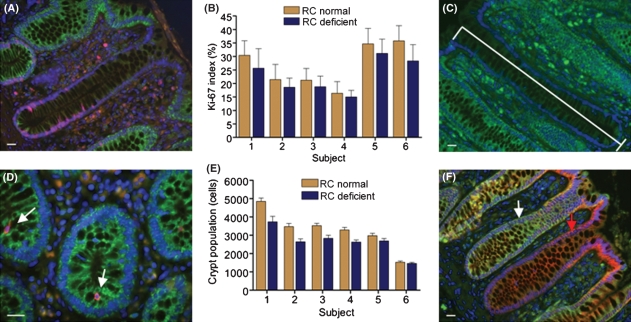
Dual immunofluorescence investigating the functional effects of respiratory chain (RC) deficiency. RC function is detected by expression of cytochrome *c* oxidase (COX) subunit I. (A) Expression of Ki-67 (red) and COX subunit I (green). A RC deficient crypt is shown in this image. (B) Frequency of actively proliferating cells in RC normal and deficient crypts ± SD. The numbers of each type of crypt analysed for each subject were: subject 1, age 79, 15 normal, five deficient; subject 2, age 89, 15 normal, 14 deficient; subject 3, age 84, 13 normal six deficient; subject 4, age 73, 15 normal, 13 deficient; subject 5, age 85, 15 normal, eight deficient; subject 6, age 75, 15 normal, ten deficient. Panel C shows a RC deficient hemi-crypt to illustrate nuclei counting to determine crypt length. (D) Expression of M30 CytoDeath (red) and COX subunit I (green), the white arrows highlight apoptotic cells. (E) Crypt cell population based on the crypt length multiplied by the average crypt circumference of the RC normal and RC deficient crypts for each subject. Subjects and numbers of crypts; same as for Ki-67 analysis. Error bars are the SEM of the products. (F) Expression of β-catenin (red) and COX subunit I (green). β-catenin was detected in the cytoplasm of both RC normal (white arrow) and RC deficient crypts (red arrow). Scale bars 20 μm.

Colorectal tumours often show altered RC function and mtDNA mutations have been detected in cell lines derived from these tumours (Polyak *et al.*, 1998). Mutations in the *APC* gene occur in ∼80% of colorectal tumours and often result in a truncated APC protein which disrupts the WNT signalling pathway. This leads to β-catenin accumulation in the nucleus causing aberrant transcription of genes regulating cell proliferation (Fearnhead *et al.*, 2001). To determine if RC deficiency was associated with aberrant WNT signalling, we studied the localization of β-catenin in 49 RC normal crypts and 22 RC deficient crypts from eight subjects aged 59–85. Uniform cytoplasmic staining of β-catenin was observed in all crypts, irrespective of RC function (Fig. 1F), indicating no effect on WNT signalling in the deficient cells.

Studies in mice with a proof-reading defect in the mtDNA polymerase, leading to an increased rate of mtDNA mutations, provide a link between high levels of mtDNA mutations and a premature aging phenotype (Trifunovic *et al.*, 2004; Kujoth *et al.*, 2005). The mtDNA mutation rate in these mice exceeds that seen in normal aging mice and humans, and there remains uncertainty as to the role of mtDNA mutations in normal human aging. In the colon RC deficiency is common, with ∼15% of crypts RC deficient by the 7th decade, caused by clonally expanded mtDNA point mutations (Taylor *et al.*, 2003). The architecture of colonic crypts provides a unique model in which to investigate some of the functional consequences of age related RC deficiency in a replicating tissue. The changes seen with decreased proliferation are entirely compatible with observations in patients with mtDNA disease. In these patients, in replicating tissues such as blood, there is selection against those cells with high levels of mtDNA mutation and the most severe biochemical defect (Rajasimha *et al.*, 2008), supporting an effect of the RC deficiency on cell proliferation and apoptosis. However, the effects of the RC deficiency observed in human colon with aging are unlikely to be limited to impaired cell proliferation and increased apoptosis. Many cellular processes within colonocytes are ATP dependent, such as electrolyte transport and mucus secretion (Kunzelmann & Mall, 2002), and thus are likely to be impaired, contributing to the aging process in these cells.

In conclusion, we have shown that RC deficiency secondary to the clonal expansion of pathogenic mtDNA mutations affects essential cellular functions in the human colonic epithelium. The changes we see are subtle; however, small quantitative changes in these essential functions may contribute to the progressive deterioration in tissue homeostasis which we commonly observe with age. RC deficiency and mtDNA mutations are a feature of aging in a number of replicating cell populations (Nekhaeva *et al.*, 2002; Shin *et al.*, 2004a,b; McDonald *et al.*, 2008; Fellous *et al.*, 2009a,b;), and while these mutations alone are unlikely to account for all of the complex phenotypes associated with aging (Kirkwood, 2008), our data may help to explain the role of mitochondrial defects in the decline in cellular self renewal which occurs during human aging.
